# Supplementary material for: Time-dependent changes in stepping performance and velocity following partial dopaminergic lesions in the SNc of male and female rats
Source: PLoS One. 2026 May 21;21(5):e0337381. doi: 10.1371/journal.pone.0337381 (PMC13193502; doi:10.1371/journal.pone.0337381)
Supplement: S1 File — (DOCX) [file pone.0337381.s001.docx]

**Supporting Information 1**

*Assessment of inter-rater agreement*

Nigral cell counts were obtained independently by two raters for each hemisphere and tissue section. Because the number of tissue sections available per animal varied, agreement was evaluated at two levels. First, agreement was assessed at the tissue level, where each observation corresponded to a specific animal, tissue section, and hemisphere. This level was used to characterize the raw measurement agreement between raters and to identify potentially discrepant tissue-level observations. Second, because the main inferential analysis treated the animal as the unit of inference, counts were averaged across tissue sections within each animal, hemisphere, and rater, and agreement was reassessed at the animal-side level. This second level was used to determine whether averaging raters was justified for the primary analysis.

Agreement between raters was quantified using two-way absolute-agreement intraclass correlation coefficients (ICC), including both single-measure and average-measure estimates. Agreement was further examined using Bland-Altman analysis to visualize the mean inter-rater difference and limits of agreement and to inspect potential systematic bias across the range of counts. To characterize the largest tissue-level disagreements, absolute inter-rater differences were ranked, and cases above the 95th percentile were examined descriptively with respect to hemisphere, treatment, and time. Final hemisphere-specific counts used in the primary analysis were defined as the mean of both raters after agreement had been evaluated at the animal-side level.

**Results**

*Inter-rater agreement at the tissue level*

At the tissue level, raters showed positive correspondence across the full range of counts, but dispersion was non-negligible and several larger discrepancies were observed. Bland-Altman inspection showed that the mean inter-rater difference was centered near zero, arguing against a marked global systematic bias, but dispersion was greater for the left hemisphere. The largest tissue-level disagreements were concentrated in the left hemisphere and occurred predominantly in lesioned animals, although they were distributed across t1, t2, and t3 rather than restricted to a single time level (Fig S1.1, Table S1.1). These findings indicated that tissue-level agreement was sufficient to proceed to aggregation but also justified reassessing agreement at the level used for the primary inferential analysis.

*Inter-rater agreement at the animal-side level*

After averaging counts across tissue sections within each animal, hemisphere, and rater, agreement was reassessed at the animal-side level. The single-measure absolute-agreement intraclass correlation coefficient was ICC(A,1) = 0.841 (95% CI: 0.741-0.904), and the average-measure estimate was ICC(A,2) = 0.913 (95% CI: 0.851-0.950). Bland-Altman analysis showed that the mean difference between raters remained centered near zero, consistent with the absence of marked systematic bias, although dispersion was still somewhat greater for the contralateral hemisphere (Fig S1.2, Table S1.1). Together, these analyses supported the use of the mean of both raters as the final hemisphere-specific count for each animal in the main analysis.

**Figure S1.1.** *Bland-Altman plot of inter-rater agreement at the tissue level*

**
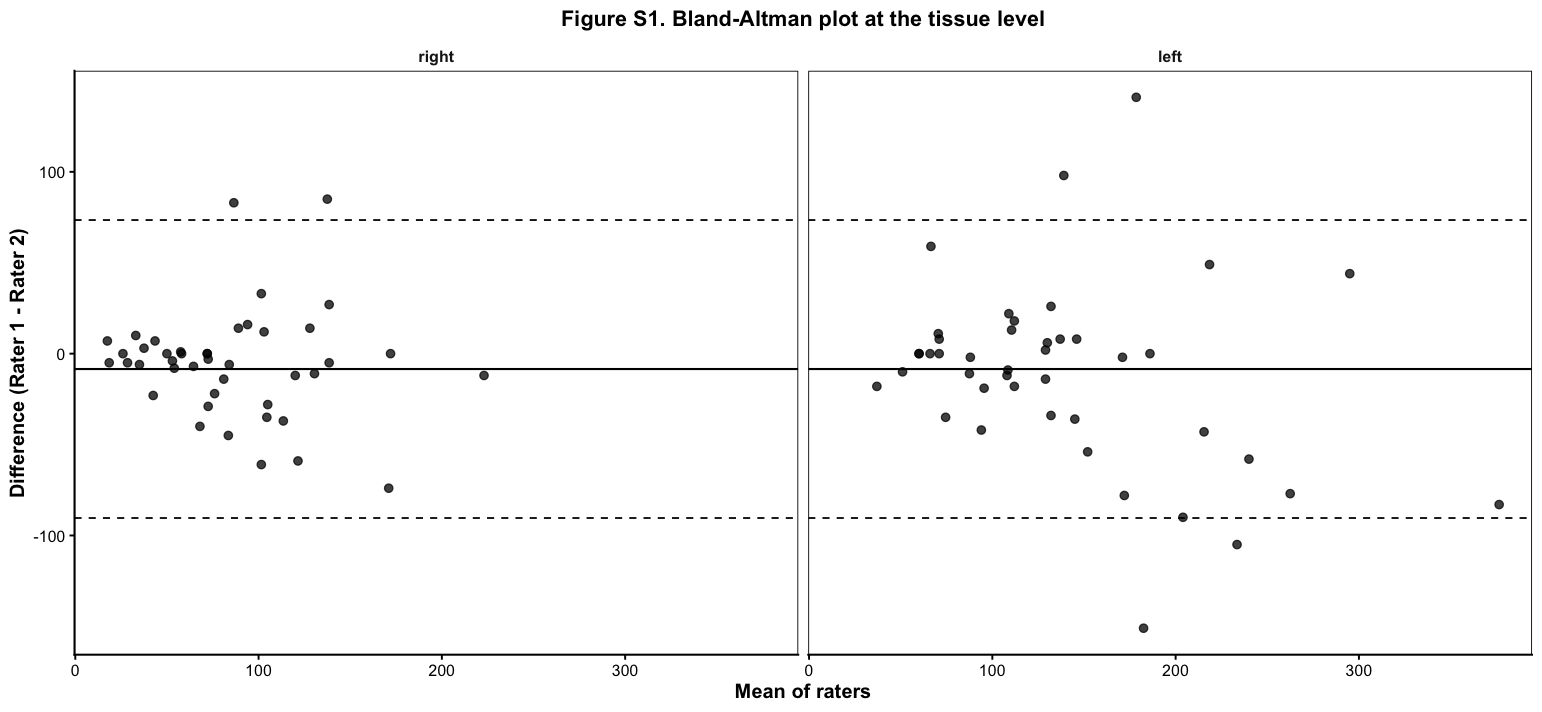
**

Bland-Altman plots showing the agreement between raters at the tissue level, stratified by hemisphere. Each point represents one tissue-specific count for one hemisphere from one animal. The solid horizontal line indicates the mean inter-rater difference, and dashed lines indicate the 95% limits of agreement. Dispersion was greater in the left hemisphere, and the largest disagreements were concentrated in a small subset of tissue-level observations.

**Figure S1.2.** *Bland-Altman plot of inter-rater agreement at the animal-side level*

**
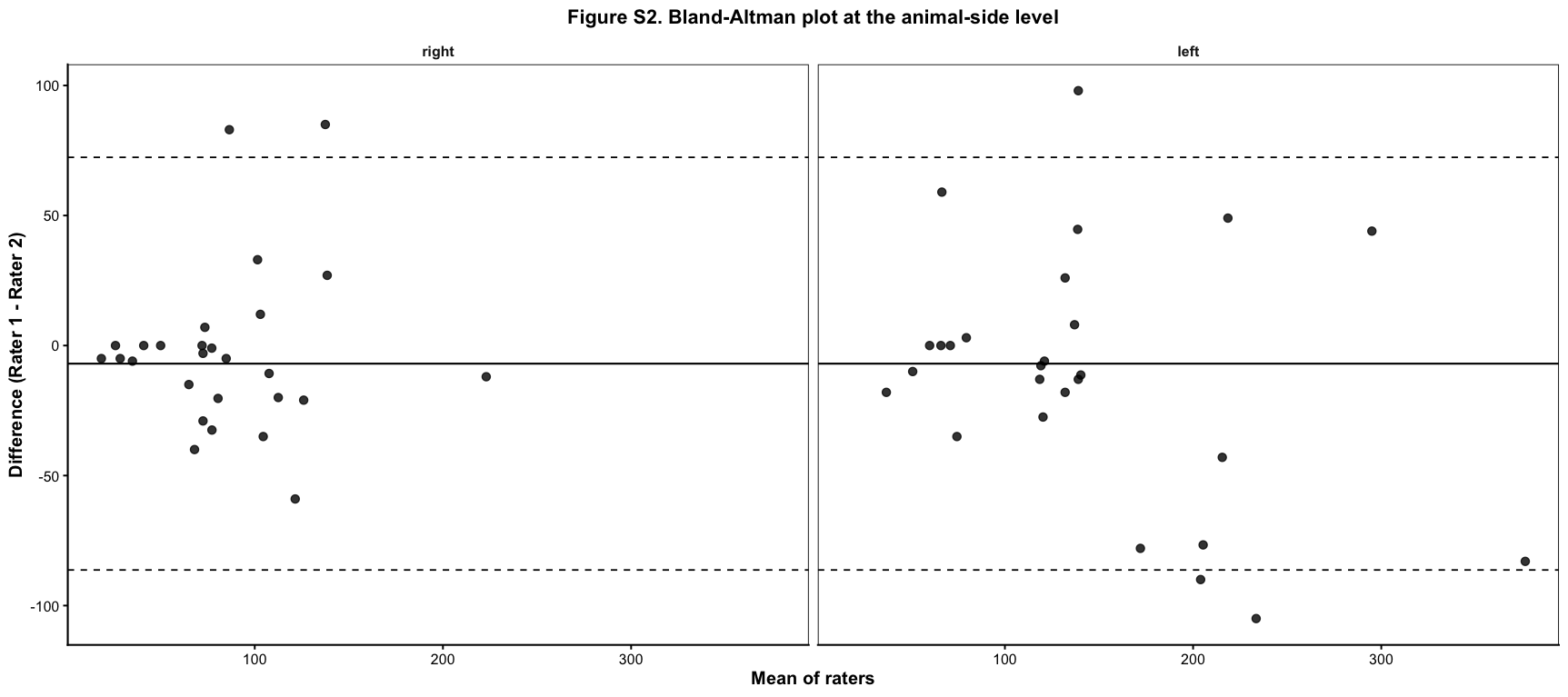
**

Bland-Altman plots showing the agreement between raters after counts were averaged across tissue sections within each animal, hemisphere, and rater. Each point represents one animal-side observation. The solid horizontal line indicates the mean inter-rater difference, and dashed lines indicate the 95% limits of agreement. Agreement improved after aggregation to the animal-side level, supporting the use of the average of both raters for the primary analysis.

**Table S1.1.** *Intraclass correlation coefficients for inter-rater agreement*

| ***Level*** | ***ICC_type*** | ***Estimate*** | ***CI_lower*** | ***CI_upper*** | ***p_value*** |
| --- | --- | --- | --- | --- | --- |
| Tissue | ICC(A,1) | 0.808 | 0.719 | 0.871 | <0.001 |
| Tissue | ICC(A,2) | 0.894 | 0.836 | 0.931 | <0.001 |
| Animal-side | ICC(A,1) | 0.841 | 0.741 | 0.904 | <0.001 |
| Animal-side | ICC(A,2) | 0.913 | 0.851 | 0.95 | <0.001 |

Two-way absolute-agreement intraclass correlation coefficients for inter-rater agreement at the tissue and animal-side levels. ICC(A,1) corresponds to the reliability of a single rater, whereas ICC(A,2) corresponds to the reliability of the average of two raters. Final inferential analyses were based on the average of both raters after aggregation to the animal-side level.
