# Supplementary material for: Time-dependent changes in stepping performance and velocity following partial dopaminergic lesions in the SNc of male and female rats
Source: PLoS One. 2026 May 21;21(5):e0337381. doi: 10.1371/journal.pone.0337381 (PMC13193502; doi:10.1371/journal.pone.0337381)
Supplement: S2 File — (DOCX) [file pone.0337381.s002.docx]

**Supporting Information 2**

**Bayesian Modeling**

As noted above, each subject’s rate of footfall errors was modeled as a Poisson process, governed by a rate parameter *θ*, which we evaluated using a hierarchical generalized linear model. As noted each subject’s error rate was modeled as a mixture of fixed effects (*β*) and a random intercept for a given subject (*γ*_s_), as previously described by Equation 1:

*μ*_s,w_ = *γ*_s_ + X_s_⋅*β*_x_ + T_s_⋅X_s_⋅*β*_tx_ + W_s_⋅*β*_w_ + T_s_⋅(W_s_⋅*β*_t_) [Eq. 1]

Here, dummy codes indicating the experimental condition (T) and week (W), turning the respective parameters in the model on and off. This is then related to the subject’s actual error rate using a log link:

*θ*_s,_*_w_* = exp(*μ_s_*_,_*_w_*) [Eq. 2]

Errors*_s_*_,_*_w_* ~ *Poisson*(*θ*_s,_*_w_*)

Our priors for all fixed effects *β* were broad, normal distributions centered on zero. Given the low expected rate of footfall errors in general, we considered these to be weakly informative:

*β* ~ *Normal*(0, 2) for all fixed effects [Eq. 3]

The one hierarchical term in the model was a random intercept

*γ* ~ *Normal*(*η*, *ψ*) for all subjects [Eq. 4]

*η* ~ *Normal*(0, 2) (prior on the intercept’s population mean)

*ψ* ~ *Exponential*(1.5) (prior on the intercept’s population standard deviation)

While our prior on the population mean *η* was broad to a degree similar to our fixed effects, we used a more informative prior on the population standard deviation *ψ*.

The asymmetry of errors was modeled by the total errors made on the subject’s left side as a binomial process, governed by a probability of left error *τ* for each error made. These were also estimated using the same overall structure to *μ_s_*_,_*_w_*, as described above (albeit different actual values for those parameters). The same regularizing priors were used as those in the Errors model specified by Equations 2 and 3.

*τ*_s,_*_w_* ~ *InverseLogit*(*μ_s_*_,_*_w_*) = (1 + exp(-*μ_s_*_,_*_w_*))^-1^ [Eq. 5]

LeftErrors*_s_*_,_*_w_* ~ *Binomial*(Errors*_s_*_,_*_w_*, *τ*_s,_*_w_*)

Finally, the velocity was modeled using a linear model after converting velocities to log units. This helped to manage the considerable positive skew of the velocity data. Residual error on log units was also treated as a fixed effect across subjects.

log(Velocity*_s_*_,_*_w_*) ~ *Normal*(*μ*_s,_*_w_*, *σ*) [Eq. 6]

*β* ~ *Normal*(0, 5) for all fixed effects

*μ* ~ *Normal*(*η*, *ψ*) for all subjects

*η* ~ *Normal*(3, 2) (prior on the intercept’s population mean)

*ψ* ~ *Exponential*(2.5) (prior on the intercept’s population standard deviation)

*σ* ~ *Exponential*(2.5) (prior on the residual error term)

For each of the three models above, parameters were fit using Hamiltonian Monte Carlo (specifically, Stan’s “No U-Turn Sampler”), allowing numerical estimation of the uncertainty of both individual parameters and of derived values (such as a subject’s expected performance during any given week). All subjects were included in a single model, contributing data from those weeks in which their performance was recorded. In each case, models were estimated using four parallel chains, each using 1000 warmup steps and 1000 sampling steps. Adapt_delta was set to 0.98, with a max_treedepth of 12.
