# Supplementary material for: Time-dependent changes in stepping performance and velocity following partial dopaminergic lesions in the SNc of male and female rats
Source: PLoS One. 2026 May 21;21(5):e0337381. doi: 10.1371/journal.pone.0337381 (PMC13193502; doi:10.1371/journal.pone.0337381)
Supplement: Attachment — Submitted filename: Response to Reviewers.pdf [file pone.0337381.s004.pdf]

April 4, 2026

To the Reviewers
PLOS One

Re: Revised submission of manuscript PONE-D-25-59811
Title: *Time-dependent changes in stepping performance and velocity following partial dopaminergic lesions in the SNc of male and female rats*

Dear Reviewers,

We thank the Academic Editor and the Reviewers for their careful reading of our manuscript and for their constructive comments. In response to those suggestions, we have revised the manuscript extensively, with substantial changes to the Methods, Results presentation, and Discussion. In particular, we clarified the experimental design and cohort structure, expanded the description of the behavioral and histological analyses, recalibrated velocity and revised the results accordingly, redesigned the figures, and reformulated the Discussion to better define the interpretive scope of the findings and to adopt a more cautious treatment of sex-related effects.

In addition, we revised the manuscript title to better reflect the refined scope of the study and to avoid overstating sex-related effects considering the revised Discussion. The revised title is: *Time-dependent changes in stepping performance and velocity following partial dopaminergic lesions in the SNc of male and female rats.*

Below, we provide a detailed point-by-point response to each comment. All changes are reflected in the revised Manuscript, and the corresponding line numbers are indicated accordingly.

Reviewer 1

Comment 1. Experimental design and cohort structure. The rationale for using three independent post-lesion cohorts (t1, t2, t3) rather than repeated measures in the same animals could be articulated more explicitly. This decision has consequences for power

and for the interpretation of trajectories. Please also report the exact sample distribution by sex × treatment × time, ideally in a table, to provide a better understanding.

We thank you for pointing this out. We have revised the Methods to clarify the rationale for using three independent post-lesion cohorts. Animals were assigned to terminal endpoints at 2 (t1), 4(t2), or 6(t3) weeks post-lesion because quantification of TH immunoreactivity required tissue collection at each interval, precluding complete within-subject follow-up across the full 6-week period. Thus, behavioral measures were obtained longitudinally within each cohort until its designated endpoint, whereas the overall post-lesion time course reflects group-level temporal patterns derived from partially overlapping cohorts. We now make this interpretive constraint explicit in the revised manuscript. In addition, to improve transparency, we have included a table reporting the exact sample distribution by sex × treatment × cohort/time point, as requested (Methods, lines 127-142; Discussion, lines 609-615).

Comment 2. Behavioral analysis would benefit from additional validation and explanation. Authors evaluate two behavioral outcomes (footfall errors and velocity). Given the capacity of DLC to extract richer gait metrics, the rationale for this restriction should be more clearly presented in the Methods. Additionally, velocity remains in pixel-based units; this limits interpretation across animals and time points. Clarifying the absence of spatial calibration and discussing its implications would improve the manuscript.

We have revised the Methods to clarify why the DeepLabCut analysis focused on velocity and footfall errors. Because videos were acquired using a single lateral camera, the analysis was restricted to measures that could be extracted consistently and interpreted reliably under these recording conditions. Accordingly, we focused on velocity and footfall errors, which captured locomotor progression and stepping accuracy, respectively. Regarding velocity units, in the revised manuscript, velocity has now been converted from pixel-based values to cm/s for all videos using a spatial calibration based on the known 4-cm distance between adjacent rungs of the apparatus. The Methods have been updated to describe this calibration procedure, and the Results

now report velocity in cm/s accordingly (Methods, lines 181-192; 295-307). We also clarify the interpretive scope of this measure in the Discussion (lines 525-532; 631-638).

Comment 3. Statistical modeling is sophisticated but needs clearer reporting. The Bayesian hierarchical model is a strength of the study. I have some concerns:

- *Equations are difficult for general readers to follow.*
- *Model diagnostics (Rhat, ESS, trace plots, posterior predictive checks) could be reported.*
- *Post-lesion weeks are modeled as categorical factors, but interpreted temporally—this mismatch needs discussion.*

Balancing the complexity of non-linear hierarchical modeling with legibility to a general audience is always tricky, and we appreciate the recognition that our modeling efforts are seen as a strength. We have made several adjustments to hopefully facilitate reader comprehension (Methods, lines 309-334). Primarily, we have moved the bulk of the model specification (priors, link functions, Stan parameters, etc.) to an appendix at the end of the text, where we have provided a bit more description of the particulars (Appendix, lines 667-709). Our hope is that this will be less disruptive to the flow of the paper for general readers.

As we now note in Tables 2 and 3, Rhat was 1.00 and ESS was at least 1000 for all parameter estimates (given 4000 posterior samples), providing plenty of confidence that we could characterize the 95% credible interval (Results, lines 382-3, 464-5). Given the number of parameters in the model, we elected not to include trace plots; we are confident our high ESS sufficiently conveys that we had adequate mixing without needing to include a figure with over two dozen panels that general readers would not know how to interpret.

Additionally, we have effectively provided a kind of posterior prediction check, at least implicitly, in the contrast between Figure 2 (which reports data for all subjects) and Figure 3 (which reports posterior mean estimates for all subjects). Given the discrete nature of Poisson data, especially when rate parameters are low, we did not feel that taking the final step of simulating data with residual error would be helpful to the reader.

The reviewers point out, correctly, that there is an evident mismatch between the model (which treats time nominally) and our linear trendlines. We have clarified in the manuscript that these trendlines are only included as a descriptive summary, reflecting gross patterns in the data post-manipulation (Results, lines 428-434). Our feeling is that most readers would trace these functions by eye anyway. Although we considered nonlinear alternatives (such as LOESS smoothers or splines), the reduction in the sample size over time makes extrapolation much riskier. Our hope is that a linear trendline, presented with the added caveats that it is included purely for descriptive purposes, will be more helpful to the reader that merely plotting the points alone.

Comment 4. Interpretation of sex differences should be more cautious. Some sex-related effects show overlapping 95% credible intervals or inconsistent patterns across variables.

Before proposing mechanistic explanations (e.g., estrogen effects), I recommend clarifying:

- *Whether the design adequately controls for estrous cycle variability.*
- *Whether sample imbalance between sexes may influence uncertainty.*
- *Which effects are robust vs. trending.*

We agree that the interpretation of sex-related effects requires greater caution. The revised Discussion distinguish more clearly between patterns that were consistently supported by the data and those that should be interpreted more tentatively. Specifically, we now state that footfall errors did not provide strong statistical support for a sex effect, and that the differences observed were expressed mainly as variability patterns rather than as clear sex-dependent motor phenotypes (Discussion, lines 537-544; 587-592). We also clarify that the study was not designed to isolate biological sources of sex-related variance, as estrous cycle was not controlled and the sample distribution across sex, treatment, and terminal cohorts may have contributed to uncertainty in some comparisons (Discussion, lines 593-602). Accordingly, mechanistic interpretations involving estrogen-related protection or sex-dependent compensatory processes are now framed as hypotheses for future targeted studies rather than as

conclusions supported directly by the present dataset. These revisions have been incorporated into the Discussion (lines 602-608).

Comment 5. DeepLabCut training and validation details concerning DeepLabCut, only 303 frames were used to train a ResNet-152 model, which is relatively small for a deep network. In order to provide a better understanding to the reader, some points could be clarified:

- *How training frames were selected*
- *Whether frames included all gait phases*
- *How model performance was evaluated*
- *Whether labeling consistency was assessed*

We have expanded the Methods to clarify the DeepLabCut training and validation procedure. Training frames were manually selected from multiple videos to capture a representative range of postures, locomotor states, and gait-related conditions observed in the dataset, including forward progression, stationary postures, slips, and hopping-like movements. In addition, frame selection was intended to sample multiple limb configurations across the locomotor cycle, including paw contact, limb lift, and forward swing, although frames were not formally categorized into predefined gait phase classes. This clarification has now been added to the Methods (lines 199-204). We also now clarify that, although the network backbone was based on ResNet-152, DeepLabCut relies on transfer learning from models pre-trained on large image datasets, which substantially reduces the number of manually annotated frames required for accurate pose estimation. In this study, 303 annotated frames were used to provide broad coverage of the observed behavioral variability (Methods, lines 271-274).

Model performance was evaluated using the mean absolute error (MAE) between predicted and manually annotated landmark positions in both training and test sets. The trained network achieved a training MAE of 3.80 px and a test MAE of 8.41 px, which decreased to 5.42 px after applying a likelihood threshold of ≥ 0.75, indicating accurate landmark prediction suitable for downstream gait analysis. We have added these values and a brief explanation of the likelihood threshold to the revised Methods. Finally, we

clarified that landmark annotations were performed using fixed labeling criteria and reviewed for consistency across frames representing different postures and locomotor conditions. Landmarks were assigned only when the corresponding body part was clearly visible and within the frame. These clarifications have now been incorporated into the revised manuscript (Methods, lines 277-285)

Comment 6. Figures. The figures are informative. I just would suggest that red–green contrasts be avoided to improve readability for color-blind readers.

We have swapped green out for tan hues and performed level checks to verify that the color scheme is interpretable even viewed as strict grayscale.

Comment 7. TH quantification methods require more detail as TH asymmetry anchors the biological interpretation, please consider to include:

- *How SNc ROIs were defined.*
- *How optical density was normalized.*
- *Whether quantification followed stereological guidelines.*
- *How hemispheric asymmetry was calculated.*

We appreciate this comment and agree that the TH quantification procedure needed to be described in greater detail, particularly because the asymmetry measure is central to the biological interpretation of the lesion. We have revised the Methods section accordingly. In the revised version, we specify how SNc sections were identified using the Paxinos and Watson rat brain atlas and how ROIs were defined in ImageJ based on atlas-guided anatomical boundaries and consistent cytoarchitectural landmarks across hemispheres and animals. We also clarify that TH quantification was based on direct manual counts of TH-positive somatic profiles within these ROIs, performed independently by two evaluators blinded to treatment condition using the Cell Counter tool in ImageJ. Fibers and diffuse staining were excluded from the analysis. We also added information on inter-rater agreement (Methods, lines 216-224; 236-256 Supplementary material 1).

We further add that optical density measurements were not used in this study, as the analysis was based on direct cell counts. Likewise, stereological methods were not applied because our goal was not to estimate absolute neuronal number, but rather to quantify relative hemispheric depletion within each animal (Methods, lines 253-256).

Finally, we now define hemispheric asymmetry explicitly using an Asymmetry Index (AI = I/C), where the injected hemisphere is expressed relative to the contralateral side, and we specify that the primary inferential outcome was the log-transformed AI analyzed as a function of treatment, time, and their interaction (Methods, lines 249-268).

Comment 8. Discussion. Consider discussing the contribution of neuroinflammatory processes to partial dopaminergic lesions. The Discussion would benefit from acknowledging that partial or subthreshold dopaminergic lesions commonly induce early neuroinflammatory responses in the model adopted, which can influence motor adaptation, synaptic remodeling, and even sex-dependent vulnerability. Including this perspective would enrich the interpretation of your findings, especially regarding variability in gait adaptation. Recent work has highlighted the functional relevance of early inflammatory modulation in partial lesion models (https://doi.org/10.3390/neuroglia6030036), which should be included. A brief paragraph contextualizing this would strengthen the mechanistic framework of the Discussion.

We agree that the contribution of neuroinflammatory processes merits acknowledgment in the context of partial dopaminergic lesion models. Although the present study did not directly assess inflammatory markers, we acknowledge that early glial and inflammatory responses have been described in toxin-based models of nigrostriatal degeneration and may shape behavioral adaptation during the initial phases of lesion development. We have revised the Discussion accordingly to incorporate this mechanistic perspective in a more explicit way. We have also cited the suggested reference, together with additional relevant literature, to place our findings within the broader context of early inflammatory modulation in 6-OHDA models (Discussion, lines 564-579).

Minor Comments

- Week/time labels (t1, t2, t3) should be standardized across all figures and tables.

- Please check for minor typographical issues (e.g., “Gonazalez”).

We apologize for the mistakes that have now been corrected

Reviewer 2

Comment 1. For each session, the authors selected the trial with the fewest stops, or the first uninterrupted crossing. This procedure could introduce a systematic selection bias considering that stopping behaviour may represent indication of motor impairment in this lesion model. This issue should be explained or addressed in the Discussion.

We thank you for raising this point. The purpose of selecting the trial with the fewest stops, or the first uninterrupted crossing when available, was to evaluate stepping accuracy and velocity under conditions of continuous locomotion. Because pauses during ladder crossing can reflect a mixture of exploratory behavior, hesitation, and task disengagement in addition to motor difficulty, trials with fewer interruptions were used to obtain a more standardized estimate of rung negotiation performance. We agree, however, that stopping behavior may also capture a meaningful component of motor impairment in this lesion model. Accordingly, we now clarify in the Methods the rationale for this trial-selection strategy and acknowledge in the Discussion that this procedure may underrepresent deficits expressed primarily as increased stopping during crossing (Methods, lines 177-184; Discussion, lines 544-550).

Comment 2. The manuscript presents two different definitions of locomotor velocity. In the behavioral task description, velocity is “calculated from the time the four paws first contacted the rungs until the head reached the end of the ladder”, whereas in the DLC-based analysis and statistical modelling, velocity is “calculated using the displacement of the tail base across frames and reported in pixels per second (px/s)”. It should be clarified which metric were used to the reposted results.

We thank you for identifying this point. We have already clarified that the results reported in the manuscript are based on a single velocity measure. In all analyses, trial duration was defined from the onset of forward movement on the ladder to the frame in which the animal reached the end of the apparatus, and distance was estimated from the horizontal displacement of the ear-base landmark across that interval. Velocity was

calculated as displacement over time and is now reported consistently in cm/s in the revised manuscript. We have therefore revised the text to eliminate the inconsistent wording in the original submission and to provide a unified definition of velocity across the task description, DeepLabCut methods, and statistical analysis sections (Methods, lines 185-193, 195-307; Results, lines 453-474).

Comment 2. In the Discussion section, the authors emphasize on sex-specific adaptive strategies and differential sensitivity to dopaminergic depletion. However, results presented do not reveal statistically supported effects of sex for footfall errors. These statements should be reformulated more cautiously in this section.

We agree that the previous Discussion overstated the interpretation of sex-related effects, particularly in relation to footfall errors. In response, we revised this section to adopt a more cautious formulation throughout. Specifically, we no longer describe the results as supporting sex-specific adaptive strategies or differential sensitivity to dopaminergic depletion in a definitive way. Instead, the revised Discussion now presents these patterns as suggestive heterogeneity in behavioral responses, while explicitly noting that footfall errors did not show statistically supported sex effects. We also clarify that the greater variability observed in lesioned females should not be interpreted as evidence of a distinct adaptation strategy, but rather as a pattern that warrants further investigation in studies specifically designed to test sex-related mechanisms. These changes have been incorporated into the Discussion (lines 537-544, 593-602).

Comment 4. The authors state that this study aims to quantify stepping errors and overall locomotor velocity rather than to perform a detailed kinematic analysis of gait. In this case it is reasonable the use of a single lateral camera at 30 fps for their behavioral analysis. However, it is noteworthy that, despite the lesion being performed in the right hemisphere, locomotor recordings were obtained from the right side of the animal. This camera placement could potentially reduce sensitivity and accuracy for detecting left paw footfall errors, which should be clarified by the authors.

We agree that the right-sided camera placement could have reduced sensitivity for detecting some left paw footfall errors, particularly subtle contralateral slips following the right-hemisphere lesion. To minimize this limitation, footfall events were assessed frame by frame and scored only when the relevant limb was clearly visible in the recorded images. We now clarify in the Methods that footfall error scoring was conducted under the constraints of a single lateral view and was intended to quantify stepping performance under those recording conditions, rather than to provide an exhaustive account of all paw placement events (Methods, lines 181-193). We also now acknowledge in the Discussion that this recording configuration may have underrepresented some left-side errors and therefore reduced sensitivity to subtle contralateral impairments. These clarifications have been incorporated into the revised manuscript (Discussion, lines 544-550, 627-638).

Minor

- Figure 1B. The images shown should be better explained in the figure caption.
- The labels C and L in the microphotographs are not defined.

We apologize for the mistakes that have now been corrected.

We again thank the Academic Editor and the Reviewers for their careful evaluation and constructive comments. We hope that the revisions made to the manuscript, together with the detailed responses provided above, adequately address the concerns raised.

Sincerely,

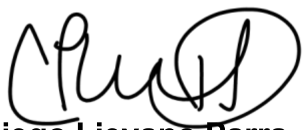

Diego Lievano Parra, Ph.D.

on behalf of all authors
